# Mechanical Strain Promotes Oligodendrocyte Differentiation by Global Changes of Gene Expression

**DOI:** 10.3389/fncel.2017.00093

**Published:** 2017-04-20

**Authors:** Anna Jagielska, Alexis L. Lowe, Ekta Makhija, Liliana Wroblewska, Jochen Guck, Robin J. M. Franklin, G. V. Shivashankar, Krystyn J. Van Vliet

**Affiliations:** ^1^Department of Materials Science and Engineering, Massachusetts Institute of TechnologyCambridge, MA, USA; ^2^Department of Neuroscience, Wellesley CollegeWellesley, MA, USA; ^3^Mechanobiology Institute, National University of SingaporeSingapore, Singapore; ^4^Department of Biological Engineering, Massachusetts Institute of TechnologyCambridge, MA, USA; ^5^Biotechnology Center, Technische Universität DresdenDresden, Germany; ^6^Wellcome Trust - Medical Research Council Cambridge Stem Cell Institute and Department of Clinical Neurosciences, University of CambridgeCambridge, UK; ^7^BioSystems and Micromechanics Inter-Disciplinary Research Group, Singapore-MIT Alliance for Research and TechnologySingapore, Singapore

**Keywords:** oligodendrocytes, oligodendrocyte precursor cell (OPC), oligodendrocyte differentiation, mechanical strain, multiple sclerosis (MS), mechanotransduction, chromatin remodeling, cell nucleus shape

## Abstract

Differentiation of oligodendrocyte progenitor cells (OPC) to oligodendrocytes and subsequent axon myelination are critical steps in vertebrate central nervous system (CNS) development and regeneration. Growing evidence supports the significance of mechanical factors in oligodendrocyte biology. Here, we explore the effect of mechanical strains within physiological range on OPC proliferation and differentiation, and strain-associated changes in chromatin structure, epigenetics, and gene expression. Sustained tensile strain of 10–15% inhibited OPC proliferation and promoted differentiation into oligodendrocytes. This response to strain required specific interactions of OPCs with extracellular matrix ligands. Applied strain induced changes in nuclear shape, chromatin organization, and resulted in enhanced histone deacetylation, consistent with increased oligodendrocyte differentiation. This response was concurrent with increased mRNA levels of the epigenetic modifier histone deacetylase Hdac11. Inhibition of HDAC proteins eliminated the strain-mediated increase of OPC differentiation, demonstrating a role of HDACs in mechanotransduction of strain to chromatin. RNA sequencing revealed global changes in gene expression associated with strain. Specifically, expression of multiple genes associated with oligodendrocyte differentiation and axon-oligodendrocyte interactions was increased, including cell surface ligands (Ncam, ephrins), cyto- and nucleo-skeleton genes (Fyn, actinins, myosin, nesprin, Sun1), transcription factors (Sox10, Zfp191, Nkx2.2), and myelin genes (Cnp, Plp, Mag). These findings show how mechanical strain can be transmitted to the nucleus to promote oligodendrocyte differentiation, and identify the global landscape of signaling pathways involved in mechanotransduction. These data provide a source of potential new therapeutic avenues to enhance OPC differentiation *in vivo*.

## Introduction

Myelination of axons by oligodendrocytes in the central nervous system (CNS) is a key distinguishing process in vertebrate development. Myelin enables faster transduction of neuronal signals, and is a critical component of axon survival (Sherman and Brophy, 2005; Nave and Werner, 2014). Inadequate myelination due to developmental disorders or insufficient myelin regeneration (remyelination) leads to profound and sustained neurological disability (Franklin and ffrench-Constant, 2008; Fancy et al., 2011). Despite intensive studies of myelination and remyelination to improve understanding and treatment of myelin disorders, this complicated process that relies on close interactions among axons and oligodendrocytes remains incompletely understood. Thus, it has remained difficult to stimulate remyelination *in vivo* for many pathological conditions including multiple sclerosis (Franklin and ffrench-Constant, 2008).

Most myelination studies focus on the biochemical regulation, including the biochemical aspects of axon-oligodendrocyte contact (Barres and Raff, 1999; Nave and Werner, 2014), whereas much less is known about the role of mechanical cues in oligodendrocyte differentiation and myelination. Recent studies provide growing evidence of mechanosensitivity of oligodendrocyte lineage cells (Rosenberg et al., 2008; Kippert et al., 2009; Jagielska et al., 2012; Franze et al., 2013; Arulmoli et al., 2015; Hernandez et al., 2016; Lourenço et al., 2016; Urbanski et al., 2016; Shimizu et al., 2017). We have demonstrated that oligodendrocyte differentiation correlates with the mechanical stiffness of underlying substrata (Jagielska et al., 2012). Within the range of brain tissue stiffness (Young's moduli ranging 0.1–1 kPa), differentiation propensity decreases with decreasing substrata stiffness, suggesting that pathological changes in the mechanical environment of the cell may affect the ability to generate or regenerate myelin sheaths. Here, we focus on a different mechanical cue, induced mechanical strain, and address the question of whether tensile strains with physiological magnitudes of 10–15% modulate oligodendrocyte proliferation and differentiation. Sources of mechanical strain *in vivo* include developmental growth (Bray, 1979, 1984; Van Essen, 1997; Smith, 2009), physiological processes such as spinal cord bending, blood and cerebrospinal fluid pulsation, and pathological conditions such as trauma, axon swelling, glial scaring, or tumor growth (Cullen et al., 2007; Fisher et al., 2007; Nikic et al., 2011; Payne et al., 2012). Related to this question is a long-standing hypothesis that axon growth (increase in length and diameter) could contribute to the control of myelin sheath length and thickness (Franklin and Hinks, 1999). In support of this hypothesis is the observation that primary developmental myelination produces a thicker and longer myelin sheath, compared to myelin formed during remyelination. Notably, axons do not grow appreciably in adult organisms. Therefore, if axon growth-induced strain (Bray, 1979; Betz et al., 2011) is a cue for OPC differentiation and associated myelin production, then the absence of such strain may affect thickness of myelin produced during remyelination in adults, in addition to the biochemical and cellular changes that also accompany stages of CNS development (Blakemore, 1974).

We find that static tensile strains within the range observed *in vivo* (10–15%) significantly decrease proliferation and increase differentiation of OPCs, and that this response is mediated by specific ligand-receptor interactions between the cell and substrata. We show that the applied strain is transferred to cell nucleus, where it alters gene expression (Dahl et al., 2008; Shivashankar, 2011; Mendez and Janmey, 2012; Graham and Burridge, 2016) in a way consistent with enhanced oligodendrocyte differentiation. Such findings prompt further consideration of the physical environments *in vivo* that may stimulate myelination, and show opportunities to engineer environments and therapies based on mechanotransduction pathways that promote remyelination.

## Materials and methods

### Ethics statement

This study was carried out in accordance with the guidelines of the National Institutes of Health for animal care and use (Guide for the Care and Use of Laboratory Animals) and the protocol was approved by the Institutional Animal Care and Use Committee at the Massachusetts Institute of Technology (MIT Committee on Animal Care).

### Cell culture and media

OPCs were isolated from mixed glial cultures obtained from Sprague Dawley rats, as described previously (McCarthy and de Vellis, 1980). Briefly, mixed glial cultures established from neonatal cortices were maintained in 10% fetal bovine serum (FBS, Atlanta Biologicals) for 10–14 days prior to overnight shaking to remove OPCs. After shake-off, OPCs were purified from microglia by differential adhesion to untreated polystyrene surface. OPCs were maintained in a progenitor state in DMEM (Gibco) with SATO's modification [5 μg/ml insulin, 50 μg/ml holo-transferrin, 5 ng/ml sodium selenate, 16.1 μg/ml putrescine, 62 ng/ml progesterone, 0.1 mg/ml bovine serum albumin (BSA), 0.4 μg/ml Tri-iodothyroxine (T3), 0.4 μg/ml L-Thyroxine (T4)] plus 10 ng/ml PDGF-A and 10 ng/ml FGF2 (Peprotech); progenitor medium. To induce differentiation, OPCs were cultured in SATO's medium without FGF2 and PDGF-A and with 0.5% fetal bovine serum (FBS, Atlanta Biologicals); differentiation medium.

### Fabrication and functionalization of elastomeric plates

Polydimethylsiloxane (PDMS) cell culture plates were fabricated from Sylgard 184 silicone (Dow Corning), using 20:1 ratio of base to curing agent. This enabled seeding of the cells onto elastomeric (stretchable) culture surfaces. The silicone mixture was degassed, poured into molds, and cured for 12 h at 45°C. Plates were removed from molds and soaked in acetone (room temperature, 12 h), to remove unreacted oligomers. After 12 h drying at 45°C, plates were soaked in ethanol for sterilization (12 h), then dried at 45°C (12 h) and stored under sterile conditions. Before cell seeding, PDMS plates were functionalized with one of the ligands: poly-D-lysine (MW: 70,000, Sigma), fibronectin (from bovine plasma, Sigma), or laminin [mouse natural laminin from Engelbreth-Holm-Swarm (EHS) sarcoma, Invitrogen], according to the following steps: plates were surface-activated in air plasma for 30 min, to make the silicone surface hydrophilic; this was followed by immediate incubation with APTES for 2 h [(3-Aminopropyl) triethoxysilane, Sigma, 100 mM, room temperature] to introduce −NH_2_ groups to the silicone surface, and washed three times with deionized water; next, plates were incubated for 4 h at room temperature with solution of molecular cross-linker BS3 (1 mM, Covachem) and a ligand (fibronectin, laminin, or poly-D-lysine, termed PDL; 50 μg/ml) in HEPES buffer (50 mM, pH 8.0), followed by three washes with PBS (phosphate buffer saline, pH 7.4). The efficiency of ligand deposition was verified in separate experiments with fluorescently labeled PDL (Sigma), fibronectin, or laminin (Cytoskeleton). OPCs were seeded on PDMS plates immediately after functionalization, at densities ~25,000 cells/cm^2^.

### Application of tensile strain to cells plated on elastomeric PDMS plates

Before strain application, OPCs were cultured on functionalized PDMS plates in progenitor media, at 37°C and 5% CO_2_ for 24 h after seeding, to ensure sufficient cell attachment to the surface. For proliferation assays, we applied biaxial static tensile strain of 15% for 24 h, using a commercial strain device (FlexCell) and commercial cell culture silicone plates (Bioflex, ligand-functionalized as described above). During these experiments, OPCs were incubated in progenitor media at 37°C and 5% CO_2_. For long-term differentiation assays, we used our custom-designed and fabricated strain devices (Zeiger, 2013), and applied 10% static tensile strain to OPCs grown on the custom-fabricated and functionalized PDMS plates as described above, for 3 or 5 days. Tensile strain of 10% was the maximum achievable with these PDMS plates, beyond which fracture within the plate material could occur during several days of static applied strain. In this assay, cells were incubated in differentiation media, at 37°C, 5% CO_2_. For both assays, the control samples were OPCs cultured on PDMS plates at the same conditions, but without applied strain (We note that repeated attempts to use the FlexCell device for multiple-day differentiation experiments were unsuccessful due to cell detachment beyond day 2.)

Stability and uniformity of strain applied with our customized strain device were assessed by phase contrast time lapse imaging of fiducial markers on the PDMS surface. Strain transferred to the plate was calculated as $\varepsilon =\frac{L-L_{0}}{L_{0}}\cdot 100\%$, where *L*_0_ is the distance between two fiducial points in the unstrained plate and *L* is the distance between those points under applied static strain. Transfer of external strain to the plate was complete, uniform at all positions (deviations of <1% strain), and stable over time (fluctuations <0.3% strain during 18 h observation, data not shown).

**Figure 1:**
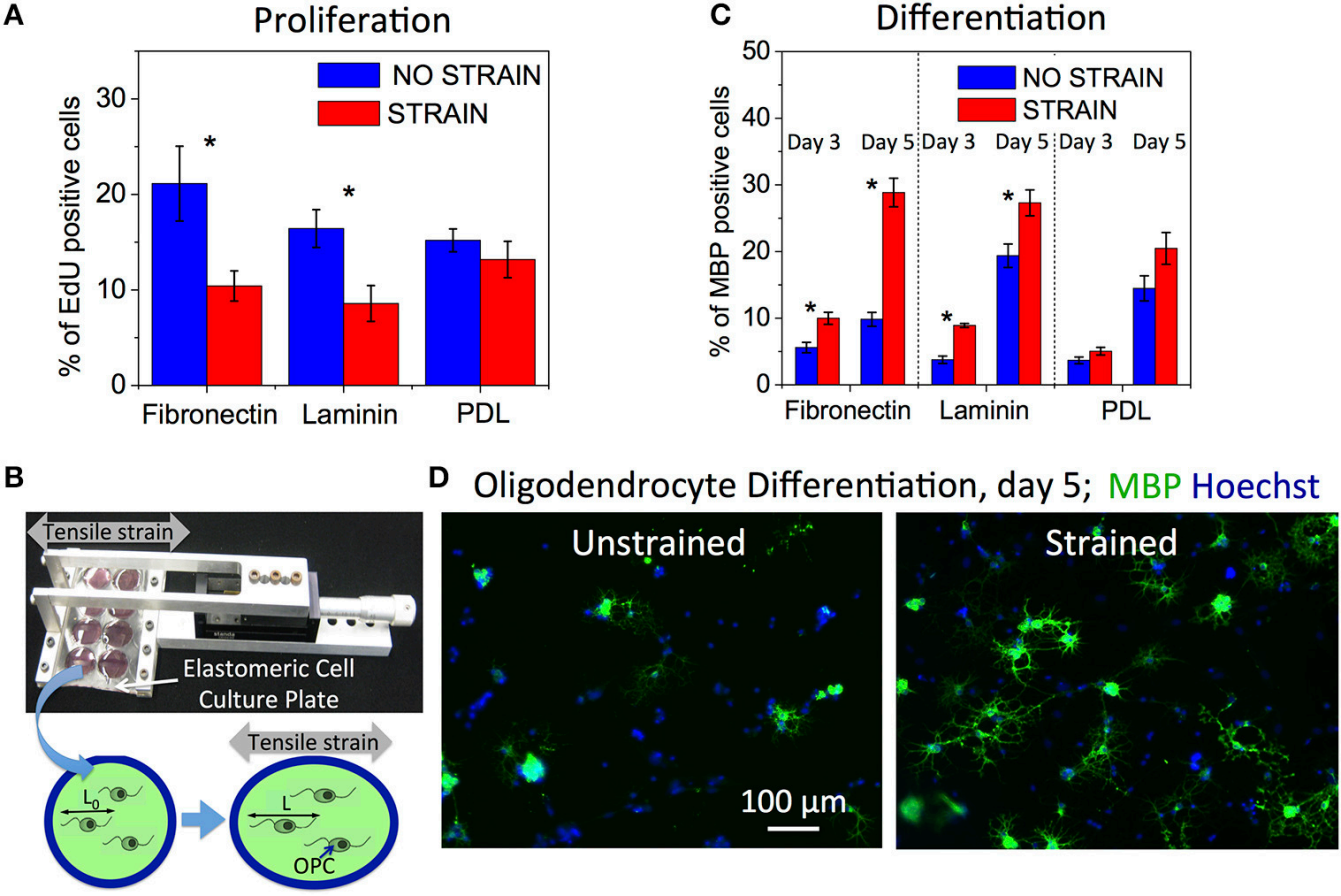
**Effect of static strain on OPC proliferation and differentiation, on fibronectin, laminin, or poly-D-lysine (PDL) coated PDMS substrates. (A)** Proliferation under static strain (15%), expressed as percentage of EdU-positive cells. **(B)** Schematic of tensile strain transfer to cells via custom stretcher that applies strain to the elastomeric plate on which cells are grown. Stretching of elastomeric plate results in elongation of cells adhered to the plate surface. **(C)** Differentiation under static strain (10%) at 3 and 5 days of strain duration, expressed as percentage of MBP-positive cells. **(D)** Representative fluorescence images of immunostaining against MBP (green) and nuclei staining (Hoechst, blue) at day 5 of differentiation, for unstrained and strained cell populations. Blue bars, unstrained samples; red bars, strained samples. *N* ≥ 6 experiments per condition; Average number of cells analyzed per condition: proliferation—8,076, differentiation—2,212; Error bars are SEM (standard error of the mean); ^*^p < 0.05.

Using a similar imaging approach, we evaluated strain transfer and stability in OPCs cultured on our customized PDMS plates, at 37°C and 5% CO_2_. Applied strain of 10% to the PDMS plate resulted in 10% strain in OPCs grown on PDMS substrata, as assessed by measuring the distance between cell process endpoints before and after strain application (Figure 1B). Strain in OPCs remained stable for at least half of the cells during a 2 h observation; the cells for which observed strain was not constant and matched to the applied strain were those that moved their processes from the initial position (data not shown). Cell somata were stretched along the applied strain axis by 10% in at least half of the cells; in the others, cell bodies contracted or remained unchanged. The initial strain on the somata remained stable for about 20 min, after which we observed increased changes in somata shape often associated with cell motility (data not shown).

### Proliferation and differentiation assays

#### Cell proliferation

*Cell proliferation* was evaluated after 24 h of applied tensile strain by EdU incorporation (ClickIt assay, Invitrogen). Live cells were incubated with EdU (10 μM) during the last 2 h of strain duration. Next, EdU containing media were removed, and OPCs were live-stained with propidium iodide (PI, Invitrogen) to mark live cells, followed by three washes in PBS. After fixing with 4% paraformaldehyde, OPCs were stained with azide-Alexa Fluor 488 (5 μM) for EdU detection, followed by nuclei staining with Hoechst (Invitrogen) to determine total number of cells. EdU-positive OPCs (cells in S-phase) were detected using fluorescence imaging and proliferation was expressed as percent of EdU-positive cells with respect to a total number of cells. Cells were summed over at least 10 distinct, imaged regions per sample. Only cells that were living before immunostaining (did not stain red with PI) were considered. At least six samples were analyzed for each condition: strain or no strain, and three different PDMS functionalizations: fibronectin, laminin, or PDL.

#### Differentiation

*Differentiation* was evaluated after 3 or 5 days of applied tensile strain to cells maintained in differentiating media, by immunostaining against myelin basic protein, MBP. Before MBP-staining, cells were first live-stained with PI to mark live cells, followed by three washes in PBS. Next, cells were fixed with 4% paraformaldehyde, immunostained against MBP, followed by staining with secondary antibody with Alexa Fluor 488 (Invitrogen), and staining nuclei with Hoechst. Differentiation was calculated as percentage of MBP-positive cells with respect to a total number of cells. Cells were summed over at least 10 distinct, imaged regions per sample. Only cells that were living before immunostaining were considered. At least six (day 3 time point) or nine (day 5 time point) samples were analyzed for each condition: strain or no strain, and three different ligand functionalizations: fibronectin, laminin, or PDL.

#### Immunocytochemistry

Cells were fixed with 4% paraformaldehyde for 20 min, washed with PBS, blocked with 1% BSA (bovine serum albumin) in PBS (blocking solution) for 30 min, and permeabilized with 0.1% Triton X100 for 3 min. Primary antibodies were diluted in blocking solution and incubated with cells at room temperature for 1 h. After three washes with PBS, cells were incubated for 1 h with secondary antibodies (diluted in PBS to final concentration 4 μg/ml). After three washes with PBS, cell nuclei were stained with Hoechst. The primary antibodies used for immunocytochemistry were rat anti-MBP (Serotec) used to measure OPC differentiation, and rabbit anti-AcH3K14 (against acetylated lysine 14 in histone H3, Millipore). Secondary antibodies were goat anti-rabbit IgG Alexa Fluor 488, and rabbit anti-rat IgG Alexa Fluor 488 (Invitrogen).

#### Optical microscopy image acquisition

Images were acquired using an inverted fluorescence microscope (Olympus IX-81) equipped with an Orca-R2 camera and a Lumen fluorescence lamp or inverted confocal microscope (Nikon) for high resolution chromatin imaging. For live imaging of nuclei in OPCs grown on PDMS substrata, we used either epifluorescence with 40x objective (Olympus LUCPlanFLN 40x. N.A. 0.60, Ph2) in an environmental chamber (37°C and 5% CO_2_) to test strain transfer to nuclei, or confocal fluorescence with 100x oil immersion lens and 4x scanning zoom to image chromatin. For fluorescence-based assays with fixed cells (proliferation, differentiation, and histone acetylation), images were acquired at 10x with Olympus IX-81, at room temperature in PBS.

#### Live nucleus and chromatin fluorescence imaging

To visualize nucleus shape in OPCs, we used the GFP-NLS—green fluorescent protein (GFP) with nuclear localization sequence (NLS). The GFP-NLS DNA construct was delivered to OPCs and oligodendrocytes via retrovirus infection. Briefly, virus was produced in HEK cells, by adding a mixture of 2 μg of DNA (virus components and GFP-NLS component), 150 μl of DMEM, and 7.5 μl of attractene (Qiagen) per 2 million of HEK cells in ~90% confluent culture. After 48 h incubation, media with released virus containing GFP-NLS DNA were collected, mixed 1:1 with cell progenitor media, and added through sterile-filter to OPC cultures. Media containing virus was removed after 16 h and cells were imaged after another 24 h. For fluorescence visualization of chromatin in live cells, we used OPCs transfected with H2B-GFP (histone 2B fused to green fluorescent protein). Briefly, CellLight H2B-GFP BacMam2 mix (Thermo Fisher Scientific) was added to cell media at 2 μL per 50,000 cells. Time-lapse fluorescence movies of H2B-GFP expressing OPCs were acquired 24 h after cell transfection with H2B-GFP gene. Fresh differentiating medium (without growth factors) was added and strain was applied to cells cultured on PDMS plates immediately before image acquisition. Movies were recorded with 30 s intervals, for a total duration of 30 min for each nucleus. Multiple subsets of several nuclei were imaged during consecutive 30 min sessions for each condition; full data sets were collected within the first 3 h of imaging and strain duration (*N* > 20 nuclei per condition). Time-lapse movies were used to obtain data about nucleus and chromatin shape, using custom procedures in Matlab and ImageJ. Nucleus rigid-body translations and rotations were removed from each data set prior to analysis.

#### Quantification of nucleus shape and chromatin organization

Nucleus shape was quantified by two shape descriptors: ***Circularity*** = ***4***π ***Area/Perimeter***^2^ and ***Solidity*** = ***Area/Convex_Area***, which range from 0 to 1, with values closer to 1 describing more circular and more solid shapes. To quantify chromatin condensation we counted number of chromatin intensity maxima corresponding to chromatin condensation regions, with amplitudes above a chosen threshold of 50 intensity units (to account only for more condensed areas), along the major and minor axis of the nucleus. The count along the axis with a larger number of maxima was then reported.

#### Chromatin acetylation assay

Chromatin acetylation of histone H3 at the lysine 14 residue (AcH3K14) was quantified with immunofluorescence, at time points of 0, 12, 24, and 48 h after inducing oligodendrocyte differentiation by removing growth factors from the media (i.e., differentiating media). Cells cultured on stretched PDMS plates and the corresponding unstrained control plates were fixed at one of the above time points, immunostained with antibody against acetylated lysine 14 in histone H3 (Millipore), and imaged using fluorescence microscope. Levels of histone acetylation were calculated as percent of AcH3K14 positive nuclei, with respect to the total number of nuclei stained with Hoechst (Invitrogen). Cells were summed over 10 separate imaged regions per sample. Four samples were analyzed for each time point and condition: strain or no strain, and three different functionalizations: fibronectin, laminin, or PDL.

#### HDAC inhibition

Quisinostat (Active Biochemicals) was added to cell media at final concentration 100 pM to inhibit HDAC function.

#### RNA sequencing and gene expression analysis

The mRNA was isolated using RNeasy Micro Plus Kit (Qiagen) from OPCs cultured on the strained (10% static tension) and unstrained (control) PDMS plates, coated with laminin, at 24 h time point after inducing differentiation via removal of growth factors from cell media (differentiating media). The mRNA samples (*N* = 3 for unstrained and *N* = 2 for strained conditions) were analyzed by RNA sequencing using Illumina HiSeq 2000 sequencer. Sequencing data were processed using bioinformatics pipeline provided by the MIT Biomicrocenter. Briefly, Illumina reads were mapped onto the rat genome (rn5) using OLego software (Wu et al., 2013), differential gene expression analysis was obtained with DESeq (Anders and Huber, 2010), and Spotfire (Tibco) was used for gene clustering. Gene mapping on signaling pathways was obtained using Ingenuity Pathway Analysis software (Qiagen).

#### RT-qPCR

Reverse transcription quantitative polymerase chain reaction (RT-qPCR) was carried out for OPCs at different time points of strain duration (12, 24, and 72 h) to compare expression of Hdac11 gene in strained and unstrained OPC samples (*N* = 3 independent samples for each condition). We also analyzed samples, which were strained 10% for 12 h and returned to 0% strain for an additional 12 h before RNA collection at 24 h after strain initiation (12/24 h). The total RNA of each sample was isolated by following the protocol for the RNeasy Mini Kit (Qiagen). RNA concentrations were measured with NanoDrop spectrophotometer (ThermoFisher), and only samples with final RNA concentrations of a minimum 6 ng/μL were used for RT-qPCR (average sample RNA concentration 14.6 ± 5.3 ng/μL). QuantiTect Reverse Transcription kits and QuantiNova SYBR Green RT-PCR kits (Qiagen) were used to convert the total RNA of samples into cDNA and prepare them for qPCR reactions. Relative gene expression levels were quantified using the LightCycler® 480 Real-Time PCR System (Roche). To obtain fold changes of gene expression between strained and unstrained OPC populations, Ct-values of technical triplicates were averaged and ΔCt were calculated with respect to GAPDH reference gene for each sample. We then calculated ΔΔCt-values between strained and unstrained samples through an all-to-all comparison method for each time point. The resulting ΔΔCt were averaged and statistical significance was tested using paired student's *t*-test against a null hypothesis of ΔΔCt of zero. Results are reported as the base-2 anti-logs of the ΔΔCt means ± *SEM*, which demonstrate the average fold change of gene expression in strained samples compared to unstrained controls at each time point (i.e., values larger than 1 indicate higher gene expression in strained samples).

### Statistical analysis of data

Reported errors are standard errors of the mean, SEM. Statistical significance analysis was conducted by one-way ANOVA followed by Bonferroni tests.

## Results

### Mechanical strain decreases OPC proliferation in a ligand-dependent manner

We quantified OPC proliferation after 24 h of sustained static tensile strain (15%, commercially available FlexCell device), via EdU incorporation that labels cells in S-phase. We observed a decrease in proliferation for the strained OPC populations (Figure 1A). The relative decrease in proliferation for strained OPCs was greater for cells grown on laminin and fibronectin-coated surfaces, as compared to cells grown on poly-D-lysine (PDL)-coated surfaces. Laminin and fibronectin are components of the extracellular matrix (ECM) in the CNS and specifically bind to cell membrane receptors, such as integrins (Buttery and ffrench-Constant, 1999; Relvas et al., 2001; Camara et al., 2009), which are known mechanotransducers (Schwartz and DeSimone, 2008); in contrast, PDL provides only non-specific interactions with the cell membrane. These results indicate the involvement of interactions between OPCs and the ECM in the mechanotransduction of tensile strain that inhibits OPC proliferation.

### Mechanical strain stimulates OPC differentiation in a ligand-dependent manner

We then assessed OPC differentiation as a function of strain after 3 and 5 days of applying static tensile strain using immunostaining against myelin basic protein (MBP), a marker of mature oligodendrocytes. To enable long-term (>24 h) cell culture under strain conditions, we used our customized elastomeric strain device (Zeiger et al., 2016, Figure 1B) and applied 10% static tensile strain (maximum strain achievable in this experimental setup, see Section Materials and Methods) to OPCs cultured in differentiating media. We observed an increased number of MBP expressing cells for the strained cell population compared to unstrained controls at both time points (Figures 1C,D). As expected, more cells had differentiated at the later time point of day 5, as compared with day 3. The differentiation increase for the strained populations was significantly larger for cells cultured on surfaces coated with fibronectin and laminin than on PDL, similar to the ligand-specific response of OPCs to proliferation.

These data suggest that tensile strain accelerates the default steps along the OPC differentiation path, which involves exiting the cell cycle followed by differentiation to mature oligodendrocytes. Differentiation of OPCs *in vivo* and *in vitro* (upon chemical induction) involves multiple epigenetic changes necessary to activate expression of myelin associated genes and other proteins involved in differentiation (Liu and Casaccia, 2010). We speculated that the external mechanical strain transduced to the nucleus (e.g., by the cytoskeleton) could modulate chromatin structure and, through these changes, promote OPC differentiation. Plausibly, mechanically-induced chemical signaling could also modify chromatin structure during the course of differentiation. To test our hypothesis, we analyzed the effect of mechanical strain on nuclear responses in OPCs.

### Mechanical strain changes nucleus shape and chromatin organization

**Figure 2:**
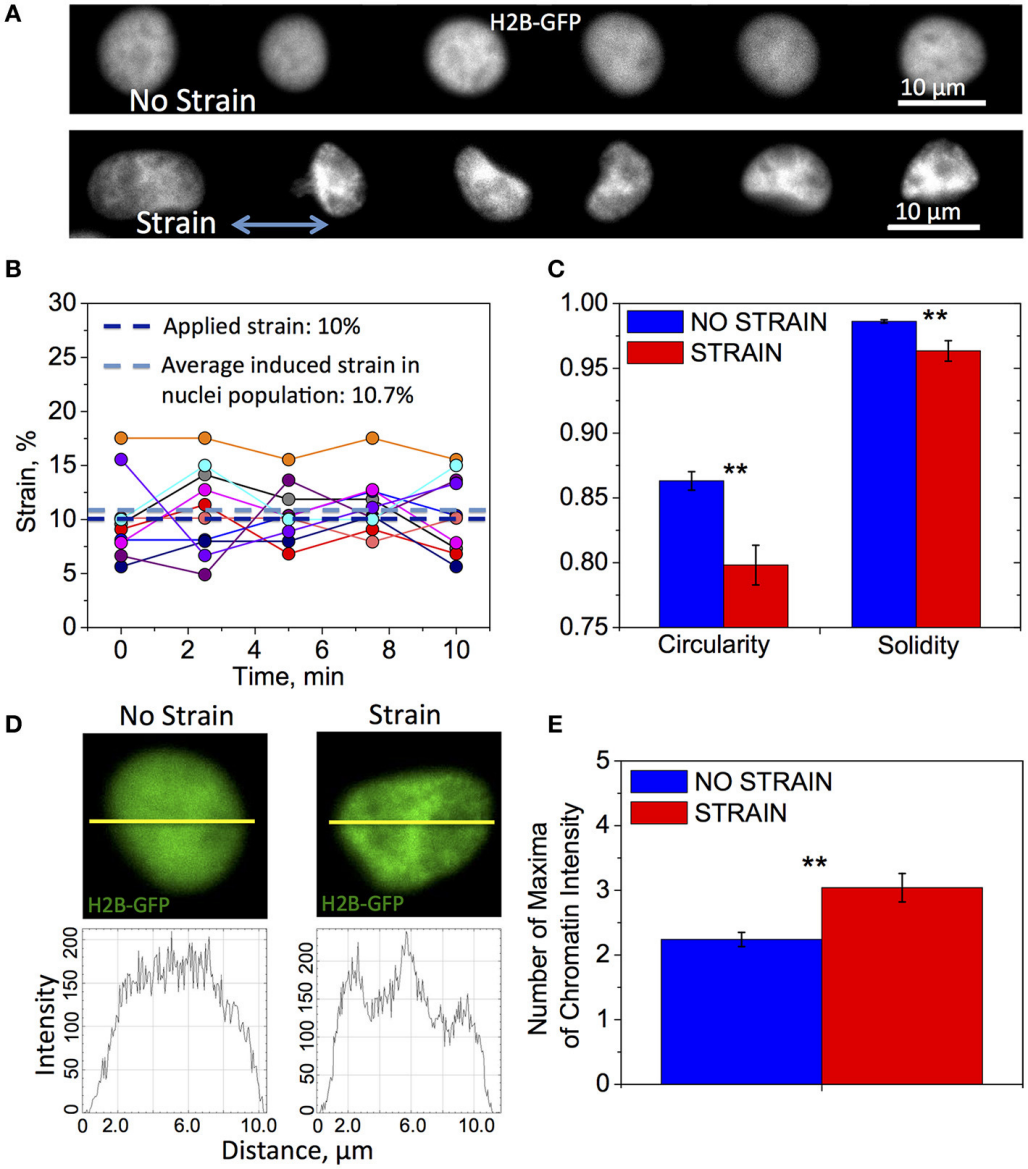
**Differences in nuclear shape and chromatin organization between unstrained and strained OPCs**. Time-lapse fluorescence images for data in subfigures **(A,C–E)** of H2B-GFP expressing nuclei in live OPCs were collected within the first 3 h of strain duration; for data in **(B)** GFP-NLS nuclei were imaged within first 10 min of strain duration. **(A)** Representative chromatin structures for unstrained (top) and strained (bottom) OPC nuclei. Blue arrow shows direction of tensile strain; **(B)** Strain (elongation) of OPC nuclei along strain directions measured during 10 min of 10% applied strain; Dark blue line, applied strain (10%); Light blue line, strain induced on nuclei, averaged over 10 analyzed nuclei (10.7%); Circles, strain exerted on nucleus at each time point; each color represents different nucleus (*N* = 10); **(C)** Average nucleus circularity and solidity for unstrained (blue) and strained (red) OPC populations; **(D)** Examples of nuclei (top) for unstrained (left) and strained (right) nuclei, and corresponding chromatin intensity profiles (bottom) along nuclear diameter (yellow line); **(E)** Chromatin condensation extent, expressed as average number of chromatin fluorescence intensity maxima along major or minor axis, for unstrained (blue) and strained (red) OPC populations. *N* > 20 nuclei per condition; Error bars are SEM (Standard error of the mean); ^**^p < 0.01.

Applied strain induced significant changes in nuclear shape and chromatin structure (Figure 2A). We examined the initial transfer of the tensile strain to the cell nucleus in live OPCs by measuring its elongation parallel to direction of strain, before and after applying 10% strain to the PDMS culture plate. A set of the same fluorescent nuclei expressing GFP-NLS (*N* = 10) was imaged for 10 min, first in unstrained and then strained conditions. We observed an average 10% elongation of the nuclear diameter, with respect to the unstrained nuclei of the same cells (Figure 2B). This nuclear strain exhibited small fluctuations about the mean value (Figure 2B). Although more extended visualization of the nuclei was not possible via this method due to significant photobleaching, these results show that applied strain rapidly resulted in nucleus shape change sustained during 10 min observation.

Next, we examined the effect of strain on chromatin organization and nucleus shape within the first 3 h of strain duration, using high magnification confocal time-lapse imaging of nuclei expressing H2B-GFP. In these experiments, the sets of nuclei imaged under strained and unstrained conditions were distinct and each nucleus within the condition subset was imaged over 30 min. We observed significant differences in nuclear shape characterized by circularity and solidity between strained and unstrained nuclei (see Section Materials and Methods, Quantification of nucleus shape and chromatin organization). Nuclei in strained cells exhibited lower average circularity and solidity values, indicating a more elongated and “ruffled” shape (Figures 2A,C). We also observed differences in chromatin organization inside the nucleus. Nuclei in strained cells exhibited more condensed chromatin (Figure 2A, “strain”), as quantified by the number of chromatin intensity maxima (defined in Section Materials and Methods, Quantification of nucleus shape and chromatin organization; Figures 2D,E).

### Mechanical strain increases histone deacetylation, consistent with OPC differentiation

**Figure 3:**
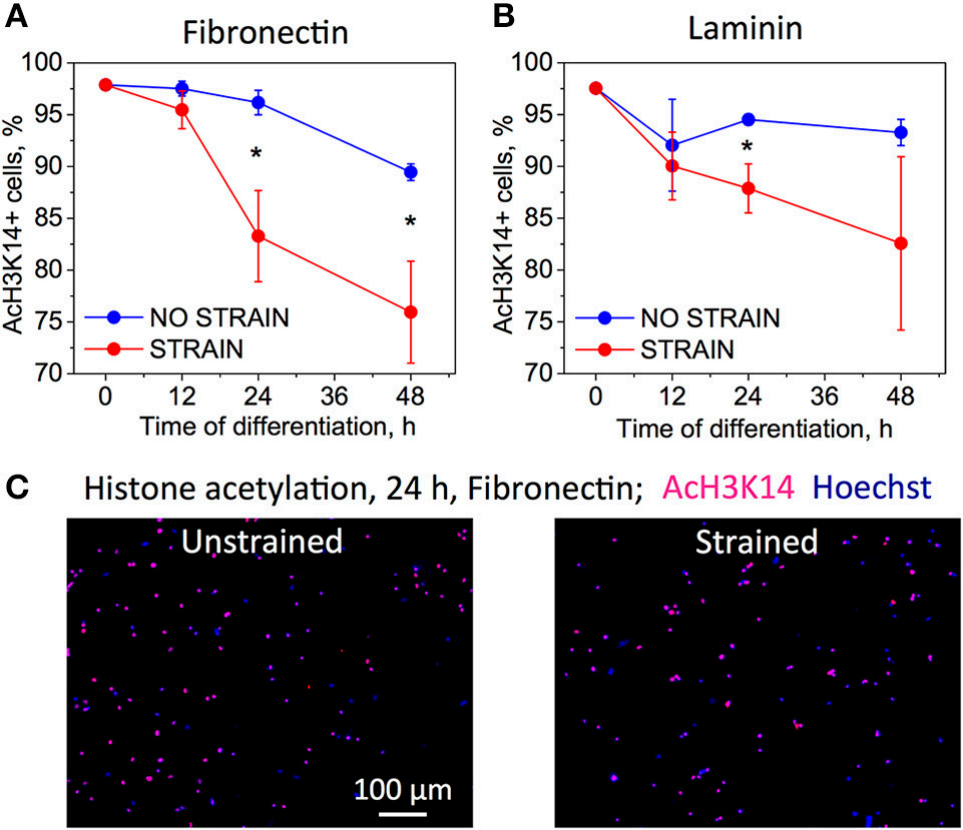
**Effect of strain on histone acetylation in OPCs on (A)** fibronectin, and **(B)** laminin coated PDMS substrates, for time points 0–48 h of strain duration and incubation in differentiating media. Acetylation expressed as percentage of cells that immunostained against acetyl group on histone 3, lysine 14 residue (AcH3K14). Blue bars, unstrained samples; red bars, strained samples (static strain of 10%); **(C)** Examples of fluorescence images quantified in **(A,B)** for unstrained and strained OPCs, at 24 h strain duration on fibronectin; blue, nuclear staining with Hoechst; pink, overlap with red color staining for AcH3K14. *N* = 4 experiments per timepoint; Average number of cells analyzed per timepoint: 1,069; Error bars are *SEM* (Standard error of the mean). ^*^p < 0.05.

To analyze the effect of strain on epigenetic modifications, we immunostained chromatin against acetylated lysine 14 in histone 3 (AcH3K14) for both strained and unstrained OPC populations. Decreased acetylation in this location was demonstrated previously to correlate with progression of chemically induced OPC differentiation in unstrained cell cultures (Liu et al., 2009; Liu and Casaccia, 2010). OPCs cultured in differentiating media, on laminin or fibronectin-coated PDMS plates (ligands for which we observed increased OPC differentiation under strain) were subjected to 10% static tensile strain using our customized strain device and immunostained at multiple time points of applied strain: 0, 12, 24, and 48 h. Unstrained control cells cultured in the same conditions were stained at the same time points. We observed a stronger decay of acetylation within this timeframe for strained cells for both ligands (Figure 3). This result is consistent with the observed increase in OPC differentiation under strain.

### HDACs are involved in strain-mediated OPC differentiation

**Figure 4:**
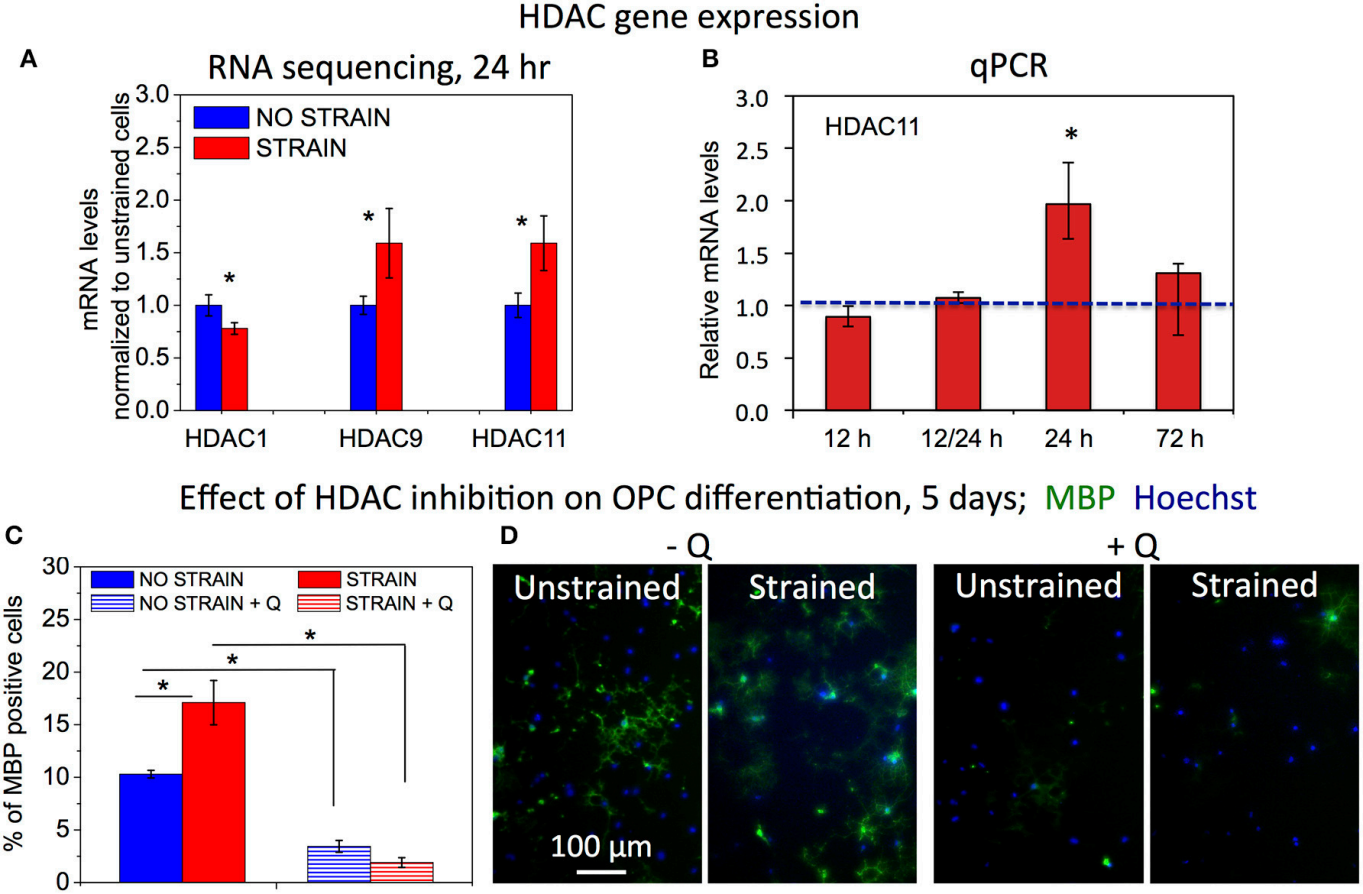
**Effect of HDAC inhibition on strain-mediated OPC differentiation. (A)** mRNA levels of histone deacetylases (HDACs) for unstrained (blue) and strained (red) OPC populations (normalized by values for unstrained cell populations), obtained from Illumina RNA sequencing; mRNA collected after 24 h of strain (10%) applied to cells cultured on laminin coated PDMS plates. *N* = 2 for strained and *N* = 3 for unstrained samples. **(B)** mRNA levels of Hdac11 in strained OPCs (red) relative to unstrained controls (normalized to 1—blue line), obtained from qPCR for different durations of applied static strain (10%): 12 h, 12/24 h—12 h strain followed by 12 h strain release (RNA levels tested at total of 24 h after strain initiation), 24 h, and 72 h. Hdac11 gene expression was significantly higher in strained samples after 24 h of applied strain (*p* = 0.004), in agreement with RNA sequencing data. *N* = 3 independent samples per condition. **(C)** Effect of HDAC inhibition with Quisinostat on OPC differentiation measured after 5 days of strain (10%) applied to cells cultured on laminin coated PDMS plates, and expressed as percentage of MBP-positive cells. Blue, unstrained, untreated sample; blue stripes, unstrained sample, treated with Quisinostat (Q, 100 pM); red, strained, untreated sample; red stripes, strained sample, treated with Quisinostat. **(D)** Examples of fluorescence images quantified in **(C)** for unstrained and strained OPCs, without (−Q) and with (+Q) Quisinostat; blue, nuclear staining with Hoechst; green, immunostaining against MBP. For **(C,D)** *N* ≥ 2 samples per condition; for **(C,D)** average number of cells analyzed per condition: 1,130; error bars are SEM (Standard error of the mean); ^*^p < 0.05.

Decreased histone acetylation in strained OPCs suggests increased activity of histone deacetylases (HDACs) under strained conditions. Consistent with this result, our RNA sequencing data (discussed in more detail in the next section of the Results) showed increased gene expression for Hdac9 and Hdac11 in strained cell populations, measured at 24 h of strain duration (Figure 4A). Increased expression of Hdac11 gene in strained cells was further confirmed with real time PCR (Figure 4B). Using qPCR, we compared mRNA levels of Hdac11 in strained OPCs and unstrained controls at different time points of strain duration, for 12, 12/24 (12 h strain followed by 12 h of strain release, and RNA collection at 24 h after strain initiation), 24, and 72 h. Relative increase of Hdac11 levels in strained samples was the highest at 24 h of strain duration. In contrast, expression of Hdac1 was slightly lower in strained cells (Figure 4A). To test whether HDACs are involved in the mechanotransduction of strain to the cell nucleus and chromatin, we compared the effect of strain on OPC differentiation in cell cultures treated with Quisinostat, a potent pharmacological HDAC inhibitor, with respect to untreated controls. Cells grown on laminin-coated PDMS plates with applied 10% strain and unstrained controls were cultured in differentiating media containing Quisinostat or the same media without Quisinostat, and immunostained against MBP after 5 days. Untreated cells under strained conditions exhibited a higher percentage of MBP+ cells (Figures 4B,C: left “−Q'). HDAC inhibition with Quisinostat significantly decreased OPC differentiation in both strained and unstrained conditions. This HDAC inhibition also abrogated the effect of strain on OPC differentiation (Figures 4B,C: right “+Q”), as indicated by a similarly low percentage of MBP+ cells under both strained and unstrained conditions (no statistically significant difference). This result suggests that HDACs are involved in the mechanotransduction of strain in the OPC nucleus.

### Effect of mechanical strain on gene expression associated with oligodendrocyte differentiation

To gain a broader understanding of genes and signaling pathways involved in the mechanotransduction of strain in OPCs and the resulting increase of cell differentiation, we analyzed gene transcription in strained and unstrained cell populations, using Illumina RNA sequencing. The mRNA was extracted from OPCs incubated in differentiating media and grown on PDMS substrates coated with laminin, with 10% tensile strain applied for 24 h. Genes differentially expressed between strained and unstrained cells with adjusted *p* < 0.05 were considered statistically significant and used in further analysis. There were 3,804 genes differentially expressed between strained and unstrained cells, with 1,784 up-regulated (523 with more than two-fold increase of expression level) and 2,020 down-regulated (639 with more than two-fold decrease in expression level) in strained cell populations.

**Figure 5:**
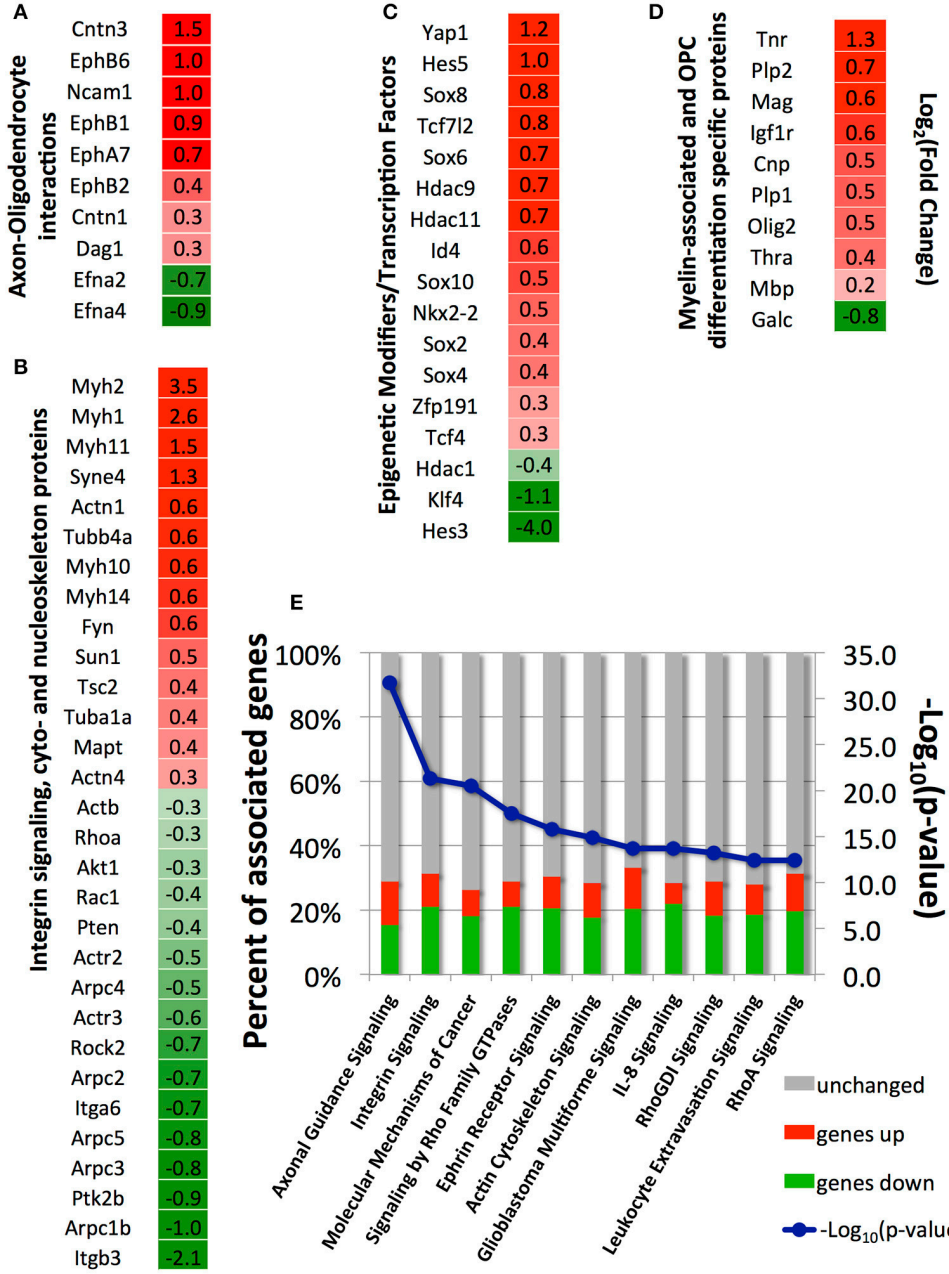
**Selected differentially expressed genes between strained and unstrained OPC populations. (A)** Genes involved in axon-oligodendrocyte interactions; **(B)** Genes involved in integrin signaling and cyto- and nucleo-skeleton remodeling; **(C)** Epigenetic modifiers and transcription factors; **(D)** Genes associated with myelin and OPC differentiation markers. **(A–D)** Column 1—gene_id. Column 2—log_2_ (fold-change), where fold-change is a ratio of mean gene expression level for stretched OPC samples to the mean gene expression level of control (unstrained) OPC samples; Color scale: red, genes upregulated in stretched samples [positive log_2_ (fold-change) values]; intensity increases with increasing relative expression level in stretched samples; green, genes downregulated in stretched samples [negative log_2_ (fold-change) values]; intensity increases with decreasing relative expression level in stretched samples. **(E)** Top canonical pathways (lowest *p*-value) associated with differentially expressed genes identified by Ingenuity Pathway analysis; blue, –log_10_(*p*-value); bars, percent of upregulated (red), downregulated (green), and unchanged (gray) genes in stretched samples with respect to unstrained control; Number of independent samples: *N* = 3 for unstrained and *N* = 2 for strained conditions.

Here, we selected four groups of genes: (A) genes involved in axon-oligodendrocyte interactions; (B) genes involved in integrin-mediated mechanotransduction and cyto- and nucleo-skeleton remodeling, (C) transcription factors and epigenetic modifiers involved in OPC differentiation; (D) genes associated with myelin and OPC differentiation (Figures 5A–D). A full list of annotated genes and expression levels, and results of the signaling pathways analysis, are provided in the Supplementary Tables 1–3.

### Major biological functions and signaling pathways influenced by mechanical strain

We used the Ingenuity Pathway Analysis software to map genes differentially expressed between strained and unstrained OPC populations on signaling pathways and biological functions. A total of 294 canonical pathways were identified to be affected by the change in gene expression with *p* < 0.05. Consistent with our analysis of individual genes described above, the most affected pathways (with lowest *p*-values) included axon guidance, integrin signaling, ephrin receptor signaling, Rho-GTPases signaling, and actin cytoskeleton signaling (Figure 5E). Other affected pathways included interleukin signaling, leukocyte extravasation signaling, B-cell receptor signaling, and cancer signaling (Figure 5E). Generally, this analysis indicates down-regulated expression of immune response contributors upon strain, which also promotes OPC differentiation (Supplementary Image 1).

## Discussion

Mechanical environment influences various aspects of cell biology, including proliferation, migration, cell survival, activation, and differentiation, and it is essential in tissue formation and stratification. Tissue stiffness has been shown to regulate biology of neurons and glia (Flanagan et al., 2002; Georges et al., 2006; Lu et al., 2006, 2010; Saha et al., 2008; Kippert et al., 2009; Christ et al., 2010; Moshayedi et al., 2010, 2014; Franze et al., 2011, 2013; Jagielska et al., 2012; Franze, 2013; Lourenço et al., 2016; Urbanski et al., 2016). In the CNS, the effect of strain has been studied primarily in the context of traumatic brain and spinal cord injury resulting from mechanical impact, and focused on associated damage to neurons (Bain et al., 2001; Smith et al., 2003; Engel et al., 2005; Cloots et al., 2008). Previous studies have shown that neurons respond to tensile and shear strains (Bray, 1979; LaPlaca et al., 1997, 2005; Lindqvist et al., 2010) and demonstrated the role of mechanical tension in axon growth (Bray, 1979, 1984; Franze et al., 2009, 2013; Smith, 2009; Franze and Guck, 2010; Betz et al., 2011) and vesicle transport in neurons (Ahmed et al., 2013). Relatively less is known about the effect of mechanical strain on function of glial cells under physiological conditions and in the pathological microenvironments in neurological diseases. Cullen et al. demonstrated that strain can induce reactive astrogliosis and cell death in neuronal-astrocytic co-cultures (Cullen et al., 2007), and Arulmoli et al. recently demonstrated the effect of strain on lineage choice during differentiation of neural stem cells (Arulmoli et al., 2015). Recent work of Poitelon et al. demonstrates that mechanical strain regulates radial sorting and subsequent myelination of peripheral axons by Schwann cells (Poitelon et al., 2016). Rosenberg et al. and recently Hernandez et al. showed that spatial constrains can increase OPC differentiation and that acute compressive strain increased chromatin condensation in OPCs (Rosenberg et al., 2008; Hernandez et al., 2016). Recently, Shimizu et al. reported increase of focal adhesions area, concurrent with YAP translocation to the nucleus in OPCs subjected to mechanical strain (Shimizu et al., 2017). This work focuses on currently unknown effects of tensile strain on proliferation and differentiation of OPCs into myelin-producing oligodendrocytes, which is required for developmental myelination and remyelination.

Increase of axon length and diameter during axon growth plausibly generate strains on the engaged oligodendrocyte. Neuronal growth cones in the CNS neurons generate protrusion forces of ~100 pN (Fuhs et al., 2013) and stresses of ~30 Pa that exert strains of 5–10% on polyacrylamide substrata with elastic modulus of 300 Pa (Betz et al., 2011); these strains are comparable to the magnitude applied to OPCs in the present study (10–15%). Engagement of cell processes with multiple axons and myelin wrapping are also likely to exert significant strain on both the oligodendrocyte and the axons. Although these specific strains have not yet been measured, the large morphological changes during oligodendrocyte differentiation and myelination, involving large cytoskeleton rearrangements (Song et al., 2001; Sherman and Brophy, 2005; Bauer et al., 2009; Kippert et al., 2009; Wang et al., 2012; Zuchero et al., 2015), suggest existence of tensile strain during these processes. Axon swelling often accompanying inflammatory processes, which can lead to significant diameter enlargement (~100%, Fisher et al., 2007), could be another source of strain exerted on oligodendrocytes adhered to those axons.

**Figure 6:**
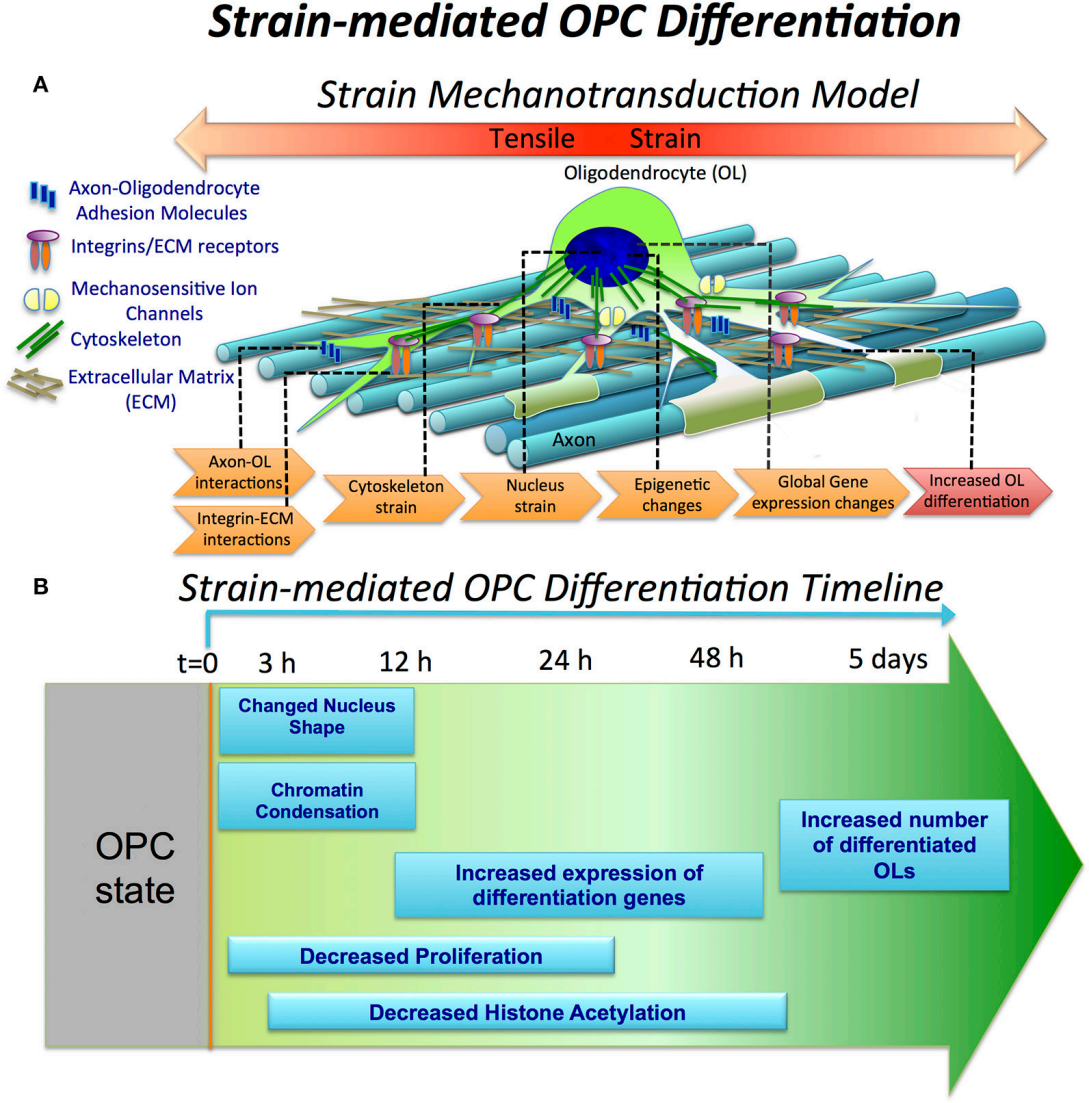
**Strain-mediated OPC differentiation. (A)** Hypothetical model of strain mechanotransduction in OPCs. Based on our data, applied tensile strain is transferred via axon-OPC and ECM-OPC interaction interfaces to OPC cytoskeleton, and then to the cell nucleus, where it changes chromatin structure and epigenetic regulation, resulting in global changes in gene expression and increased OPC differentiation. **(B)** Schematic of OPC response to tensile strain, as a function of differentiation time and strain duration. Observations are expressed as relative to the unstrained OPC nuclei. OPC differentiation (*t* = 0) is initiated by removal of growth factors from both strained and unstrained OPC cultures and concurrent application of static tensile strain to “strained” cell population. Within the first 3 h, nucleus elongation and chromatin condensation are observed in strained nuclei. From *t* = 12 to 48 h, histone H3 deacetylation increases (lysine 14). This is correlated with upregulation of many differentiation genes measured at *t* = 24 h. OPC proliferation is inhibited under strain, quantified at *t* = 24 h in non-differentiation media (containing growth factors). At extended strain duration of *t* = 5 days, a greater number of MBP-positive cells, indicating increased OPC differentiation, is observed.

Here, we investigated the effect of tensile strain on processes associated with oligodendrocyte differentiation at different time points of strain duration and differentiation progression, as summarized schematically in Figures 6A,B.

Mechanical signals can be transferred via various mechanisms from the extracellular environment to the nucleus (e.g., by integrins, mechanosensitive ion channels, or physical tension across the connected cyto- and nucleo-skeleton). This transduction of strain can alter gene expression and resulting cell differentiation (Shivashankar, 2011; Mammoto et al., 2012). Lineage commitment often involves decreased mobility and increased compaction of chromatin, which defines DNA regions accessible for transcription (Jaenisch and Bird, 2003; Hubner and Spector, 2010; Milstein and Meiners, 2011; Shivashankar, 2011; Chalut et al., 2012). Here, we demonstrated that tensile strain applied via elastomeric substrata caused elongation of intact cell nuclei and increased condensation of chromatin within first 3 h of strain duration (Figures 2, 6), consistent with differentiation progression. This was followed by increased deacetylation of histones, which was gradually growing with increasing strain duration, from 12 to 48 h (Figures 3, 6). This was consistent with elevated mRNA levels of histone deacetylases Hdac9 and Hdac11 in strained cells. Inhibition of HDAC proteins with Quisinostat eliminated the effect of strain on OPC differentiation (Figure 4), indicating that HDAC epigenetic modifiers are involved in mechanotransduction of strain in OPCs.

Consistent with observed changes in epigenetic modifications, up-regulation of multiple myelin associated genes and OPC differentiation markers, as well as many transcription factors involved in OPC differentiation (analyzed after 24 h of strain duration) further confirmed our hypothesis that strain-induced oligodendrocyte differentiation was driven by transcriptional changes (Figure 5). Analysis of the effect of differentially expressed genes on biological function indicated increased oligodendrocyte differentiation and myelination under strained conditions, consistent with our data (Supplementary Image 1).

Global changes of gene expression in OPCs induced by strain support a model of mechanotransduction in which strain is transferred to OPCs from the extracellular environment via cell-cell and cell-ECM interactions, then into the cytoskeleton, and further to the cell nucleus. This transmission results in epigenetic changes and altered gene expression consistent with differentiation (Figure 6A). Below, we briefly discuss genes involved in these stages of strain mechanotransduction, based on our RNA sequencing data.

### Genes involved in axon-oligodendrocyte interactions

Axon-oligodendrocyte interactions mediated by membrane receptors are likely an origin of strain transduction in these cells *in vivo*. We observed elevated mRNA levels of some membrane molecules, including Ncam1 (neural cell adhesion molecule), contactins (Cntn1, Cntn3), dystroglycan (Dag1), and several ephrin receptors in strained cell populations (Figure 5A). NCAM1 protein is expressed on both axonal and oligodendrocyte surfaces and it is upregulated in differentiated oligodendrocytes. Homodimer NCAM1-NCAM1 interactions between axon and OPC was suggested to play an active role in OPC differentiation (Palser et al., 2009). Contactin is another membrane protein involved in axon-oligodendrocyte interactions via binding to L1 protein on the axonal membrane. The contactin/L1 complex participates in OPC differentiation through mediating integrin β1 interaction with FYN, and through enabling local translation of Mbp gene in oligodendrocytes (Laursen et al., 2009). Increased mRNA levels of contactin 1 and 3 (Cntn1, Cntn3, Figure 5A) in strained OPCs suggest their increased interactions with axons. Several ephrin receptor genes and their ligands, ephrins, were differentially expressed between strained and unstrained populations. Specifically, we measured increased transcript levels of ephrin receptors EphA7, EphB1, EphB2, and EphB6, and downregulation of ephrin A2 and A4 (Efna2, Efna4; Figure 5A). Interactions of ephrins and ephrin receptors regulate axon myelination by oligodendrocytes (Linneberg et al., 2015). Thus, it is plausible that strain-induced changes in expression of these proteins in OPCs may influence binding to and interaction with axons during myelination. Strain-induced upregulation of cell-cell and cell-matrix adhesion genes supports the possibility that strain exerted on oligodendrocyte by axon growth can regulate oligodendrocyte differentiation and myelination.

### Genes involved in ECM, integrin signaling, cyto- and nucleo-skeleton remodeling

Mechanotransduction of external mechanical cues in many adherent cell types is initiated by interactions of transmembrane integrins with ECM ligands at the cell-ECM interface (Schwartz and DeSimone, 2008). Integrins expressed at the protein level in OPCs and oligodendrocytes include αv, α6, β3, β5, and β8. Interactions of integrin β1 with laminin have been shown to strongly affect OPC differentiation and myelination (Milner et al., 1997; Buttery and ffrench-Constant, 1999; Relvas et al., 2001; Camara et al., 2009). We found that only integrin α6 (Itga6), a receptor for fibronectin, was differentially expressed and down-regulated in strained samples. However, dystroglycan, a laminin receptor shown to increase OPC differentiation (Colognato et al., 2007; Dag1, Figure 5A), exhibited increased mRNA levels in strained populations. Expression of major ECM genes laminin (Lama, Lamb) and fibronectin (Fn1) was significantly increased in the strained cell population, suggesting an increased deposition of these ECM proteins by OPCs under strain conditions (Supplementary Table 1).

Mechanotransduction downstream of ECM-integrin interactions often involves *FYN-RhoA-ROCK-II* signaling, which controls remodeling of cytoskeleton (Amano et al., 2010). This pathway is also a strong regulator of OPC differentiation (Baer et al., 2009). Depletion of FYN was shown to impair OPC differentiation (Osterhout et al., 1999; Colognato et al., 2004; Baer et al., 2009), whereas inhibition of either RhoA or ROCK-II increased OPC differentiation (Wang et al., 2008, 2012; Baer et al., 2009; Kippert et al., 2009; Rusielewicz et al., 2014; Urbanski et al., 2016). We observed increased levels of Fyn mRNA in strained cells, and decreased transcript levels of RhoA and Rock-II genes (Fyn, Rhoa, Rock2, Figure 5B), which is consistent with observed increased OPC differentiation in stretched samples.

Many cytoskeletal genes were differentially expressed in strained samples. We observed downregulation of actin genes (Actb, Figure 5B), but increased expression of tubulin (Tuba1a and Tubb4a, Figure 5B) and tubulin-associated protein Tau genes (Mapt, Figure 5B) in strained samples. Tubulin-built microtubule cytoskeleton together with actin microfilaments are strongly remodeled during OPC differentiation to form a multi-processed, branched morphology (Song et al., 2001). Because microtubules, similarly to actin filaments, are directly connected to nuclear lamina via LINC complex (linker of nucleoskeleton and cytoskeleton; Roux et al., 2009; Graham and Burridge, 2016) and regulate nucleus shape and positioning, it is possible that tensile strain in OPCs is transferred to the nucleus predominantly via microtubule cytoskeleton.

As evidence of strain transfer to cell nucleus, we found a strong effect of tensile strain on chromatin shape and epigenetic remodeling. Consistent with these observations was increased expression of nuclear envelope genes Sun1 and Syne4 (Nesprin 4; Figure 5B), major components of LINC complex, which is a key mechanoregulator of nuclear shape and deformability (Wang et al., 2009; Chambliss et al., 2013; Makhija et al., 2016). Upregulation of Syne4 together with upregulation of tubulin genes (discussed above) further supports our hypothesis that the tensile strain in OPCs could be transferred to the nucleus predominantly via microtubules. We note that SYNE1, which binds nuclear lamina to actin filaments, was recently shown to be involved in mechanotransduction of compressive strain in OPCs (Hernandez et al., 2016).

### Transcription factors and epigenetic modifiers

Consistent with strain transduction to the nucleus, we observed changes in expression of multiple transcription factors and chromatin remodeling genes involved in OPC differentiation (Figure 5C). This included histone deacetylases HDAC9 and HDAC11. HDAC11 was shown to decrease acetylation of histone 3 at lysine 14 residues and activate transcription of Mbp and Plp genes during oligodendrocyte differentiation (Liu et al., 2009). We observed increased Hdac11 mRNA levels in strained cell populations compared to unstrained controls (Hdac11, Figure 5C). This is consistent with the comparatively sharper acetylation decline of H3K14 in strained samples (Figure 3), and with the increased expression of Plp and Mbp genes (Figure 5D). Other transcription factors required for OPC differentiation, including Sox 10, Sox 8, Nkx2.2, and Zfp191 (Chew and Gallo, 2009; Liu and Casaccia, 2010; Fancy et al., 2011), were also elevated in the strained populations (Figure 5C). We observed increased levels of the Yap1 gene in strained OPCs (Figure 5C). We further validated this result by immunostaining against YAP protein, which showed higher percentage of YAP positive cells in strained samples (Supplementary Image 2). YAP/TAZ are important mechanotransduction players in many cell types (Dupont et al., 2011; Driscoll et al., 2015) and were recently shown as essential for radial axon sorting and myelination by Schwannn cells (Poitelon et al., 2016). Consistent with our findings, recent data published during the revision of this manuscript indicated involvement of YAP in response of oligodendrocytes to material stiffness (Urbanski et al., 2016) and mechanical strain (Shimizu et al., 2017).

### Oligodendrocyte differentiation markers

Applied mechanical strain resulted in significantly higher mRNA expression of multiple OPC differentiation markers and myelin associated proteins (Figure 5D), including Cnp (myelin associated 2′,3′-Cyclic-nucleotide 3′-phosphodiesterase), Mag (myelin associated glycoprotein), Plp1 (proteolipid protein 1), Tnr (tenascin R), Igfr-I (insulin-like growth factor receptor I), and Thra (thyroid hormone receptor alpha) in strained cell populations. Mbp (myelin basic protein) also exhibited higher mRNA levels in strained cell populations, but this difference did not reach statistical significance at the 24 h time point (*p*-adjusted = 0.2). Recall that MBP protein had higher average expression in strained cell populations at days 3 and 5 of applied strain (Figures 1C,D). In general, higher mRNA expression levels of multiple differentiation markers in strained samples at 24 h correlated with the increased OPC differentiation at later timepoints (days 3 and 5).

Mechanical strain was initially transferred through strained PDMS plates to all the cells, with relative nucleus elongation, chromatin compaction, and histone deacetylation observed over minutes to hours after strain application. However, not all OPCs maintained this initial tension over several days. Some cells exhibited movement of processes and migration within ~20 min after strain initiation (data not shown). A future avenue of exploration is whether strain must be exerted for extended durations, or if only transient strain is sufficient to initiate differentiation progression. At least certain forms of transient strains appear to inhibit OPC proliferation: we found that cyclic strain applied to OPCs (data not shown), which repeatedly stretches cells by supplying a stimulus at 0.25 Hz or every 4 s, resulted in a similar inhibition of OPC proliferation as applying static strain (Figure 1A). This may suggest that a brief strain stimulus is sufficient to trigger proliferation inhibition and differentiation progression, and that cells will continue along this path even after the strain is removed.

Notably, the effect of strain on OPC proliferation and differentiation was observed only for cells grown on fibronectin and laminin, but not on bio-inert poly-D-lysine, indicating the role of specific cell-ECM interactions in mechanotransduction of applied strain in OPCs (Figure 1). The extent of proliferation decrease and differentiation increase was approximately doubled for both ECM ligands, suggesting that strain effects could be robust independent of relative fibronectin/laminin content in the ECM. This finding is notable in that ECM composition is often modified at *in vivo* inflammatory lesions, with relative overexpression of fibronectin (Sobel and Mitchell, 1989; Schregel et al., 2012; Stoffels et al., 2013; Harlow and Macklin, 2014). The role of specific interactions of cell membrane receptors with ECM ligands was also observed in strain-mediated differentiation of neural stem cells (Arulmoli et al., 2015) and the response of OPCs to material stiffness (Lourenço et al., 2016). The dependence of mechanotransduction on interactions with ECM ligands, fibronectin and laminin, further suggests involvement of the Rho/ROCK pathway in strain transduction in OPCs, through integrin-ECM binding.

These results demonstrate that mechanical tensile strain exerted on OPCs can significantly promote oligodendrocyte differentiation. Changes in axonal diameter and length during axon growth have been hypothesized to directly influence the length and thickness of myelin sheath produced by oligodendrocytes. This hypothesis derives from the observation that during organism development there is a correlation between axonal development and sheath thickness, which is absent during remyelination (Franklin and Hinks, 1999). It is conceivable that strains associated with axon growth exerted on an oligodendrocyte may stimulate its differentiation and regulate myelin thickness and length. Our findings of increased oligodendrocyte differentiation, together with upregulation of genes involved in axon-oligodendrocyte adhesion in strained cell populations, support this intriguing possibility.

In pathological microenvironments such as demyelinating lesions, integrity of axonal networks and ECM structure may be altered from normal conditions, resulting in changed tension exterted on OPCs and oligodendrocytes. Similarilly, axon swelling in these areas may provide different strains to adhered cells. Such local changes of strains in diseased tissue could contribute to altered ability of OPCs to differentiate and myelinate axons.

The genome-wide analysis of strain-induced gene expression provided herein will serve as a source of information for future studies that further explore mechanisms relating mechanical cues to myelination. From an engineering perspective, externally applied strains could be designed to stimulate OPC differentiation. Thus, our work highlights a new way of addressing the challenge of therapeutic enhancement of remyelination (Kuhlmann et al., 2008). More broadly, the pathways identified herein may provide new targets to pharmacologically promote OPC differentiation, even in the absence of applied mechanical strain.

## Author contributions

All authors listed have made substantial, direct and intellectual contribution to the work, and approved it for publication.

## Funding

We gratefully acknowledge funding from the National Multiple Sclerosis Society (RG4855A1/1), the Human Frontiers Science Program (RGP0015/2009-C), and the National Research Foundation of Singapore through the Singapore-MIT Alliance for Research and Technology (SMART), BioSystems and Micromechanics (BioSyM) interdisciplinary research group.

### Conflict of interest statement

The authors declare that the research was conducted in the absence of any commercial or financial relationships that could be construed as a potential conflict of interest.

## Supplementary material

Supplementary Table 1 **Results of Lumina RNA sequencing for strained (stretch) and unstrained (control) OPCs**.

Supplementary Table 2 **Ingenuity Canonical Pathways**.

Supplementary Table 3 **Diseases and Functions Annotation**.
