# Supplementary material for: Mechanical Strain Promotes Oligodendrocyte Differentiation by Global Changes of Gene Expression
Source: Front Cell Neurosci. 2017 Apr 20;11:93. doi: 10.3389/fncel.2017.00093 (PMC5397481; doi:10.3389/fncel.2017.00093)
Supplement: Supplementary file 4 [file Image1.PDF]

Supplementary Figure S1

a

Molecular and Cellular Function	p-value
Cellular Growth and Proliferation	6.85E-78 - 1.44E-09
Cellular Movement	9.57E-67 - 3.25E-09
Cellular Assembly and Organization	6.11E-60 - 3.26E-09
Cellular Function and Maintenance	6.11E-60 - 3.67E-09
Cell Death and Survival	3.65E-57 - 3.67E-09

Physiological System Development and Function	p-value
Tissue Morphology	4.48E-49 - 3.23E-09
Hematological System Development and Function	3.81E-46 - 3.67E-09
Organismal Survival	1.85E-45 - 1.90E-09
Immune Cell Trafficking	2.34E-42 - 1.82E-09
Nervous System Development and Function	2.59E-32 - 3.26E-09

Diseases and Disorders	p-value
Cancer	1.00E-41 - 3.75E-09
Cardiovascular Disease	1.91E-33 - 4.91E-10
Inflammatory Response	2.33E-31 - 3.67E-09
Infectious Disease	6.25E-28 - 2.41E-09
Connective Tissue Disorders	6.33E-24 - 5.67E-11

b

Nervous System Development and Function

	Activation z-score
synaptogenesis	2.7
development of neurons	1.8
neuritogenesis	1.4
development of central nervous system	1.2
myelination	1.2
quantity of neuroglia	1.1
formation of brain	1.1
proliferation of neuronal cells	0.9
outgrowth of neurons	0.9
migration of neuroglia	0.8
branching of neurons	0.8
growth of neurites	0.8
outgrowth of neurites	0.6
branching of neurites	0.5
axonogenesis	0.5
morphogenesis of neurons	0.4
morphogenesis of neurites	0.4
differentiation of neuroglia	0.4
formation of neurites	0.1
differentiation of neurons	-0.4
morphology of neuroglia	-0.8
morphology of nervous tissue	-1.2
morphology of neurons	-1.4
morphology of cerebral cortex	-1.9
abnormal morphology of cerebral cortex	-1.9
abnormal morphology of nervous system	-2.9
morphology of forebrain	-3.0
morphology of telencephalon	-3.0
abnormal morphology of forebrain	-3.0
abnormal morphology of central nervous system	-3.2
morphology of brain	-3.3
morphology of nervous system	-3.3
morphology of central nervous system	-3.7

c

Immune Cell Trafficking

	Activation z-score
accumulation of lymphocytes	-1.2
accumulation of mononuclear leukocytes	-1.4
accumulation of phagocytes	-2.0
migration of antigen presenting cells	-2.1
accumulation of myeloid cells	-2.1
activation of antigen presenting cells	-2.2
accumulation of leukocytes	-2.3
activation of phagocytes	-2.4
activation of T lymphocytes	-2.9
infiltration of leukocytes	-3.0
cell movement of macrophages	-3.1
activation of leukocytes	-3.4
adhesion of T lymphocytes	-3.4
adhesion of lymphocytes	-3.5
cell movement of antigen presenting cells	-3.5
migration of myeloid cells	-3.6
adhesion of mononuclear leukocytes	-3.6
homing of mononuclear leukocytes	-3.7
activation of mononuclear leukocytes	-3.7
migration of phagocytes	-3.9
activation of lymphocytes	-4.1
cell movement of T lymphocytes	-4.4
chemotaxis of phagocytes	-4.4
chemotaxis of myeloid cells	-4.7
T cell migration	-4.8
homing of neutrophils	-5.0
chemotaxis of neutrophils	-5.0
recruitment of myeloid cells	-5.1
chemotaxis of leukocytes	-5.3
cell movement of mononuclear leukocytes	-5.4
homing of granulocytes	-5.4
chemotaxis of granulocytes	-5.4
cell movement of lymphocytes	-5.4
recruitment of phagocytes	-5.4
recruitment of granulocytes	-5.5
migration of mononuclear leukocytes	-5.5
Lymphocyte migration	-5.5
homing of leukocytes	-5.7
recruitment of leukocytes	-5.7
recruitment of neutrophils	-5.8
cell movement of myeloid cells	-5.8
cell movement of phagocytes	-6.0
cell movement of granulocytes	-6.0
cell movement of neutrophils	-6.1
cell movement of leukocytes	-6.6
leukocyte migration	-6.9

Figure S1. ***Biological functions and diseases associated with differentially expressed genes between strained and unstrained OPCs.*** (a) Top physiological cell function categories (lowest p-value) associated with differentially expressed genes identified by Ingenuity Pathway analysis; bars correspond to $-\log_{10}$(p-value). Biological functions within (b) Nervous System Development and Function, and (c) Immune Cell Trafficking categories. Orange – positive activation z-score, indicates expected increase of function, blue - negative activation z-score, indicates expected decrease of function.
