# Supplementary material for: Mechanical Strain Promotes Oligodendrocyte Differentiation by Global Changes of Gene Expression
Source: Front Cell Neurosci. 2017 Apr 20;11:93. doi: 10.3389/fncel.2017.00093 (PMC5397481; doi:10.3389/fncel.2017.00093)
Supplement: Supplementary file 5 [file Image2.pdf]

Supplementary Figure S2

Methods and Results.

YAP1 expression in strained and unstrained OPCs was quantified by immunostaining after 30 min of applied 10% uniaxial static strain, for cells cultured on fibronectin and laminin coated (50 µg/ml) elastomeric PDMS plates (PDMS – polydimethylsiloxane). Cells were fixed in 4% paraformaldehyde, permeabilized in 0.01% Triton, then immunostained with anti-YAP1 primary antibody (Stem Cell Technologies), followed by staining with secondary antibody (AlexaFluor-549) and Hoechst. OPC populations subjected to strain exhibited more YAP1 positive cells (Fig. S2).

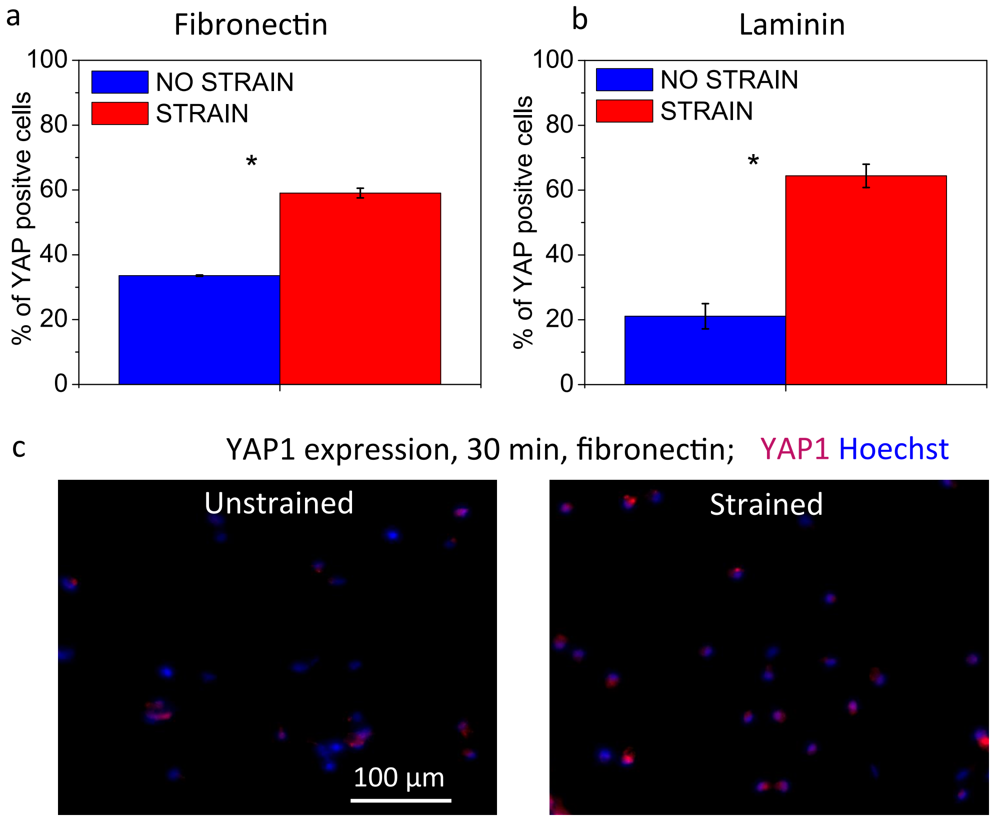

Figure S2. ***Expression of YAP1 protein in strained and unstrained OPCs.*** Percentage of YAP1 positive OPCs cultured on PDMS plates coated with a) fibronectin and b) laminin, for strained (red) and unstrained cell populations, measured after 30 min of applied static strain (10%). c) Examples of fluorescence images quantified in a). N = 2 independent samples per condition; * - p-value < 0.05, error bars are standard errors of the mean (SEM). Statistical significance analysis was conducted by one-way ANOVA followed by Bonferroni test.
